# Rectovaginal Colonization with Serotypes of Group B *Streptococci* with Reduced Penicillin Susceptibility among Pregnant Women in León, Nicaragua

**DOI:** 10.3390/pathogens11040415

**Published:** 2022-03-29

**Authors:** Teresa Alemán, Nadja A. Vielot, Roberto Herrera, Reymundo Velasquez, Tatiana Berrios, Christian Toval-Ruíz, Evert Téllez, Andres Herrera, Samir Aguilar, Sylvia Becker-Dreps, Neil French, Samuel Vilchez

**Affiliations:** 1 Center of Infectious Diseases, Department of Microbiology and Parasitology, Faculty of Medical Sciences, National Autonomous University of Nicaragua, León 00068, Nicaragua; teresa.aleman@cm.unanleon.edu.ni (T.A.); robertojhgarcia@gmail.com (R.H.); reymundo.velasquez@cm.unanleon.edu.ni (R.V.); tatianaberrios86@gmail.com (T.B.); chris0412toval@gmail.com (C.T.-R.); samuel.vilchez@cm.unanleon.edu.ni (S.V.); 2 Center for Demographic and Health Research, Faculty of Medical Sciences, National Autonomous University of Nicaragua, León 00068, Nicaragua; evert.tellez@cm.unanleon.edu.ni (E.T.); andres.herrera@cm.unanleon.edu.ni (A.H.); 3 Department of Family Medicine, University of North Carolina at Chapel Hill, Chapel Hill, NC 27599, USA; sbd@email.unc.edu; 4 Local Comprehensive Health Care System (SILAIS), Department of León, León 00068, Nicaragua; samiraguilar@yahoo.com; 5 Department of Epidemiology, University of North Carolina at Chapel Hill, Chapel Hill, NC 27516, USA; 6 Institute of Infection Veterinary & Ecological Science, University of Liverpool, Liverpool CH64 7TE, UK; n.french@liverpool.ac.uk

**Keywords:** group B *Streptococcus*, antimicrobial resistance, vaccines, Nicaragua

## Abstract

Group B *Streptococci* (GBS) are important causes of neonatal sepsis and meningitis globally. To elucidate the potential benefits of maternal GBS vaccines, data is needed on the epidemiology of maternal GBS rectovaginal colonization, distribution of serotypes, and resistance to intrapartum antibiotic prophylaxis (IAP). We collected rectal and vaginal samples from 305 pregnant women in León, Nicaragua between 35 and 40 weeks gestation. Samples were cultured for GBS and confirmed using latex agglutination. GBS isolates underwent serotyping by quantitative polymerase chain reaction, and antimicrobial susceptibility testing by disk diffusion and microdilution following Clinical Laboratory Standard Institute guidelines. Sixty-three women (20.7%) were colonized with GBS in either the rectum or the vagina. Of 91 GBS isolates collected from positive cultures, most were serotypes II (28.6%), Ia (27.5%), and III (20.9%). Most GBS isolates (52.9%) were resistant to penicillin, the first-line prophylactic antibiotic. Penicillin resistance was highly correlated with resistance to vancomycin, ceftriaxone, and meropenem. The results of our study suggest that one-fifth of pregnant women in the urban area of León, Nicaragua are colonized with GBS and risk transmitting GBS to their offspring during labor. High resistance to commonly available antibiotics in the region suggests that prophylactic maternal GBS vaccination would be an effective alternative to IAP.

## 1. Introduction

Group B *Streptococci* (GBS) disease causes an estimated 91,000 infant deaths worldwide each year [1]. Early-onset GBS disease (EOD), caused by vertical transmission from mothers who have rectal or vaginal GBS colonization, is the most common cause of neonatal sepsis in the first six days of life. Late-onset GBS disease (LOD), which occurs between days 7 and 89 of life and often presents with meningitis, is more common in preterm infants. The long-term consequences of GBS disease in infants include neurological impairment, particularly among children with LOD-associated meningitis, resulting in over 37,000 cases per year [1]. GBS screening and intrapartum antibiotic prophylaxis (IAP) is recommended in many countries for pregnant women at 36–37 weeks gestation due to its high efficacy in preventing GBS infection and EOD in neonates [2].

There are several shortcomings to IAP: first, resource-limited settings may lack laboratory capacity to perform large-scale GBS screenings; second, the increasing antimicrobial resistance of GBS strains may reduce the effectiveness of IAP; third, exposure to antibiotics during pregnancy can influence the composition of the infant gut microbiome, which is an important determinant of future disease risk [3]. Finally, IAP does not prevent LOD, which can be transmitted from mother to child after birth [4], or preterm birth, which can occur prior to GBS screening and detection [5]. In contrast, effective and safe maternal GBS vaccination can prevent maternal colonization, and can boost anti-GBS antibodies in pregnant women. These antibodies are then transferred across the placenta to the developing fetus, preventing infant GBS disease in the early and late stages while minimizing potential harm to the infant. Reduction of infant mortality is a target of the United Nations Sustainable Development Goals, and prevention of infant GBS disease can have a measurable impact toward meeting the target of 12 neonatal deaths per 1000 live births [6].

Maternal GBS vaccines are not yet licensed for use, but several candidates are under development. Most candidates have been capsular polysaccharide conjugate vaccines [7,8], which target specific capsular polysaccharides that comprise the ten known GBS serotypes; one protein subunit vaccine is currently under development [9]. Several questions remain to be answered in order to inform the development and global rollout of maternal GBS vaccines. Multivalent conjugate vaccine candidates include three to six serotypes that cause the majority of GBS cases in infants [10]. Assessment and continual monitoring of GBS serotype distribution using different methods that maximize the probability of GBS colonization in pregnant women is needed in order to ensure that vaccines will be effective against circulating serotypes in different regions. In addition, it is necessary to measure the susceptibility of GBS strains to recommended antibiotics in order to quantify the additional benefit of GBS vaccination compared to IAP, as well as the relative cost-effectiveness of GBS vaccination considering antimicrobial resistance. 

The objective of this study was to characterize GBS colonization prevalence, serotype distribution, and antimicrobial susceptibility in a sample of pregnant women in an urban area of Nicaragua, where neonatal sepsis caused over 4000 neonatal hospitalizations in 2017 [11]. Our findings will indicate whether GBS vaccine candidates would be appropriate in Nicaragua given the epidemiology of GBS, and whether they would be a viable alternative to IAP given antimicrobial susceptibility profiles. 

## 2. Results

Out of 371 pregnant women who were invited to participate during the study period, 305 (82.2%) provided informed consent to be screened for GBS colonization. Among the 305 women, the mean age was 25.1 years; 208 (92.4%) presented for screening at a gestational age of <38 weeks (as measured by last menstrual period); 117 (38.4%) were pregnant for the first time; and 176 (57.7%) had previously given live birth (Table 1). In their current pregnancy, 2 (0.7%) experienced prolonged rupture of membranes (PROM) of >18 h; 31 (10.2%) experienced risk of preterm birth; and 43 (14.1%) experienced risk of pregnancy loss (Table 1). 

Risk factors for infant GBS disease that were present in a prior pregnancy were associated with risk factors present in the current pregnancy. The strongest associations were observed between risk of preterm birth (OR: 3.6, 95% CI: 1.0, 13.1) and urinary tract infection (OR: 2.5, 95% CI: 1.1, 5.7) (Table 2). 

**Figure 1:**
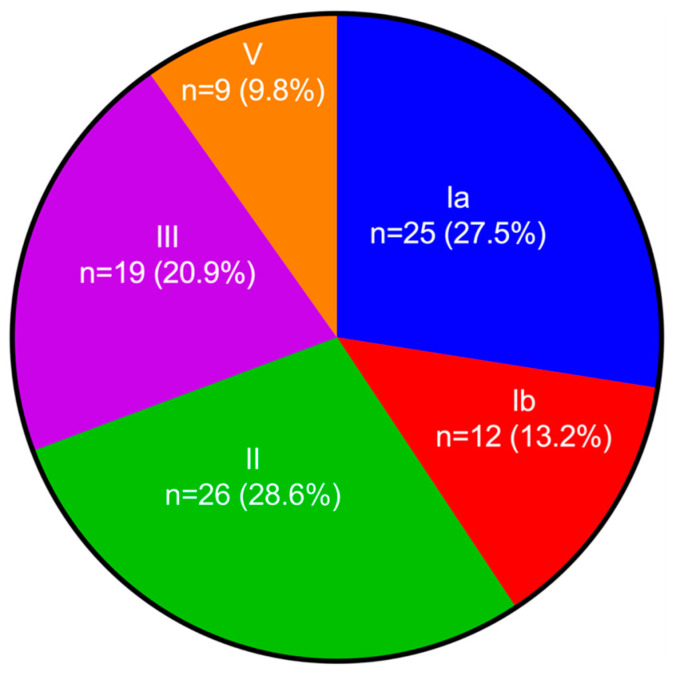
**Serotype distribution of 91 GBS isolates from 63 pregnant women from León, Nicaragua.** The total includes isolates detected in vaginal (n = 49) and rectal (n = 42) samples. Five women experienced colonization with two serotypes: Ia+III; Ib+II; Ib+III; II+III; III+V.

Sixty-three women (20.7%) were colonized with GBS in either the rectum or the vagina: 20 (31.7%) were colonized in the vagina, 15 (23.8%) in the rectum, and 28 (44.4%) in both anatomical regions. From the 63 GBS-positive women, 91 GBS isolates were recovered. Of these, 82 (90.1%) were recovered in CHROMagar™ StrepB, with or without LIM incubation. Fifty-eight (63.7%) were recovered in CNA, with or without LIM incubation (Figure S1). Five genotypes were identified: Ia; Ib; II; III; IV (Figure 1). In five women, two serotypes were identified: Ia+III; Ib+II; Ib+III; II+III; III+V. 

We were unable to determine the antimicrobial resistance profiles of six isolates due to a loss of bacterial viability. Among the remaining 85 isolates, the results of disk diffusion and microdilution assays showed that most (n = 45, 52.9%) were resistant to penicillin, the first-line antibiotic for IAP (Table 3). For validation purposes, penicillin resistance was further confirmed using e-test strips (Liofilchem^TM^ MTS^TM^) on a sample of 10 GBS isolates, as shown in Figure S2. Isolates were also resistant to clindamycin (n = 33, 38.8%), erythromycin (n = 51, 60.0%), ceftriaxone (n = 21, 24.7%), and meropenem (n = 18, 21.2%). Isolates were largely susceptible to levofloxacin, linezolid, and vancomycin. 

**Figure 2:**
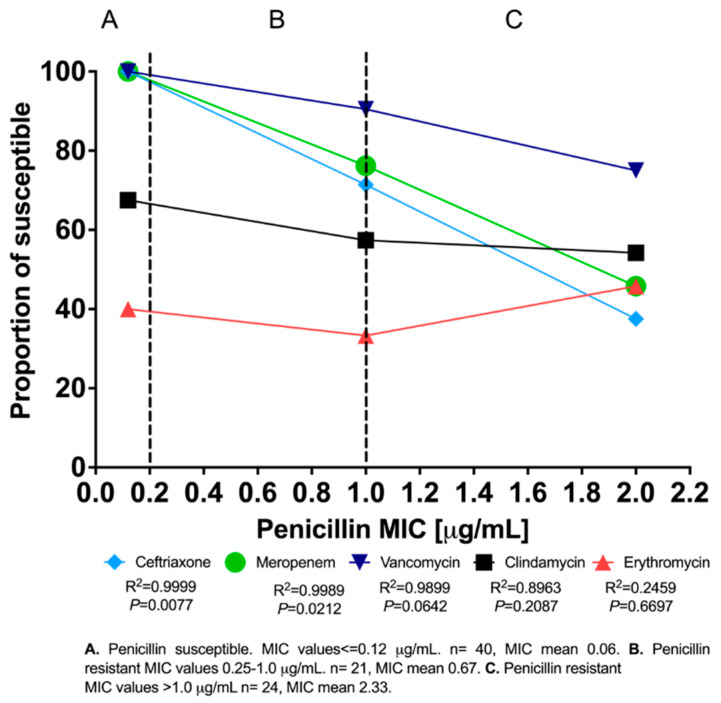
Correlation between penicillin minimum inhibitory concentrations (MICs) and susceptibility to other antibiotics in GBS isolates from pregnant women in León, Nicaragua (n = 85).

Penicillin susceptibility, as determined by minimum inhibitory concentration (MIC) values, was compared to susceptibility to ceftriaxone, meropenem, vancomycin, clindamycin, and erythromycin. Penicillin resistance was often observed alongside resistance to ceftriaxone (24.7%), meropenem (21.2%), clindamycin or erythromycin (18.8%), and vancomycin (9.4%). More specifically, penicillin susceptibility was highly correlated with susceptibility to ceftriaxone (R^2^ = 0.9999, *p* = 0.0077), meropenem (R^2^ = 0.9989, *p* = 0.0212), and vancomycin (R^2^ = 0.9899, *p* = 0.0642) (Figure 2). Where penicillin resistance was high (i.e., MIC > 1), we observed high resistance to meropenem and ceftriaxone. Clindamycin susceptibility declined slightly with increased penicillin MIC (R^2^ = 0.8963, *p* = 0.2087), but the trend plateaued and was not statistically significant. Erythromycin resistance did not show a clear pattern with respect to penicillin resistance (Figure 2). 

## 3. Discussion

In 305 pregnant women in León, Nicaragua, we observed a prevalence of rectovaginal GBS colonization of 20.7%. Risk factors for infant GBS disease were rare in the sample, but tended to be associated with a prior history of risk factors. Serotypes Ia, II, and III were predominant. 

The observed prevalence of maternal GBS colonization agrees with a prior meta-analysis of health-center-based studies from Nicaragua [12], as well as a global prevalence estimate of 18% and a preponderance of serotypes Ia, Ib, and III [13]. Scarce data from Central America estimate a maternal colonization prevalence up to 17%, with serotypes Ia and Ib accounting for nearly 70% of isolates [13]. Prophylactic polysaccharide-conjugated GBS vaccines that are currently in development include multiple serotypes to provide broad protection against circulating strains in various global regions, as monovalent vaccine candidates have not demonstrated cross-protection against other serotypes. The serotypes identified in our study sample are included in candidate pentavalent (Ia, Ib, II, III, V) and hexavalent (Ia, Ib, II, III, IV, V) vaccines, and partially in the candidate trivalent vaccine (Ia, Ib, III) [14], suggesting that various multivalent vaccine formulations could be appropriate for use in Nicaragua to reduce the burden of infant GBS disease. Protein-based vaccine candidates may protect against all GBS infections regardless of serotype; however, they are in earlier stages of development compared to polysaccharide conjugate vaccines [9,15].

Surprisingly, 52.9% of the GBS isolates were resistant to penicillin, the first-line prophylactic for women who are colonized with GBS. This finding contrasts with most other studies from around the globe, where penicillin resistant rates are between 0 and 20% [16,17,18,19]. However, prior unpublished studies from León have shown high penicillin-resistance rates based on disk diffusion testing, ranging from 23% to 94% [20,21,22,23]. Outside of Nicaragua, studies from Zimbabwe and Ethiopia have shown that 69.8% and 77.3% of GBS isolates from pregnant women were resistant to penicillin G, respectively [24,25]. Moreover, our study reports 24.7% resistance to ceftriaxone, a penicillin surrogate, which has also been documented in prior studies with resistance up to 46.5%. Genetic sequencing of GBS isolates is currently underway, and a future analysis will assess antimicrobial resistance genes and correlates of penicillin resistance in these Nicaraguan GBS strains. These studies may center on the arrangements of penicillin-binding genes that are responsible for the observed beta-lactam resistance [25,26].

In addition, our findings show that GBS isolates were also highly resistant to erythromycin and clindamycin, the first-line alternative for women with high-risk penicillin allergies. Reports from China and Zimbabwe have also demonstrated high resistance to erythromycin (77.5% and 97.7%) and clindamycin (68.3% and 55.8%), respectively [25,27,28]. The high resistance observed in our study may be due in part to the widespread over-the-counter purchase of antibiotics, including penicillin, in pharmacies. To promote antimicrobial stewardship and to reduce the improper use of antibiotics contributing to antimicrobial resistance, the enforcement of existing laws against the distribution of antibiotics without a prescription should be prioritized. In addition, studies that generate new data on antimicrobial resistance rates should be considered in the update and development of national clinical treatment guidelines. Collaboration with a central reference laboratory could advance the monitoring of antimicrobial resistance trends in the country. 

Penicillin susceptibility and corresponding MIC ranges were highly correlated with susceptibilities to other antibiotics that are largely available in Nicaragua. Prophylaxis options depend on whether the pregnant woman has a penicillin allergy, and whether GBS isolates are susceptible to clindamycin [2]. In cases of clindamycin resistance, which approached 40% in our study, vancomycin is recommended for IAP. We found high susceptibility to vancomycin even at high levels of reduced penicillin susceptibility; however, use of vancomycin for IAP is highly dependent on drug availability and known GBS status during labor. As such, prophylactic vaccination is a promising alternative to resource-intensive screening and IAP.

Until vaccines are available, cost- and resource-efficient culture methods can be used to increase GBS screening frequency in LMICs. Chromogenic media together with latex agglutination confirmation was able to identify 90% of presumptive GBS colonies. This approach can be used for high-sensitivity GBS screening in the absence of resources for proteomic or molecular confirmation. Furthermore, our findings found a correlation between a history of infant GBS risk factors and current risk factors, suggesting that women with a history of risk factors can be deprioritized for screening, and women with no known history of risk factors can be prioritized.

In Nicaragua, universal GBS screening is recommended for all pregnant women at 35–37 weeks gestation. However, screening is not universally performed due to scarce laboratory resources. Antibiotic use during labor typically results from risk-based assessments of infant GBS risk factors in the mother, such as history of having a child with GBS disease or prior evidence of GBS colonization. Cesarean section is very common in Nicaragua. Nearly one half of infants in a representative birth cohort in León were born via Cesarean section, which may result in lower infant GBS disease rates than expected (unpublished data). Ongoing data collection in León is investigating the incidence of neonatal sepsis and infant GBS disease in the public teaching hospital; however, prior data from León suggest that other bacterial species, such as *Escherichia coli*, *Klebsiella pneumoniae*, *Serratia* spp., alpha hemolytic *Streptococci*, and *Staphylococci* spp. are more commonly associated with neonatal sepsis [29,30]. 

Studies of GBS colonization could be strengthened by the use of technologies such as matrix-assisted laser desorption ionization–time of flight mass spectrometry (MALDI-TOF MS) and/or direct qPCR techniques to confirm samples that appear negative in culture [31]. However, culture and serology should remain the reference and routine techniques, particularly in the context of antimicrobial susceptibility testing. This study was conducted in the second largest city of Nicaragua and may not be generalizable to other cities in the nation nor to the Central American region. However, the frequency of GBS colonization is comparable to the average reported for other cities in Nicaragua and Central America [12,13]. Future studies are required to understand IAP implementation and effectiveness, which are imperative aspects of infant GBS disease prevention. It may also be valuable to monitor the long-term consequences of GBS, including neurodevelopmental outcomes, to understand the potential benefits of GBS vaccination in preventing a large range of negative childhood health outcomes.

## 4. Materials and Methods

### 4.1. Study Design

A prospective cross-sectional study was conducted from August 2019 through March 2020. Rosters of pregnant women who were scheduled for their third trimester antenatal care visits in the Perla Maria, Mántica Berio, and Sutiava health sectors in León, Nicaragua were provided by the local Ministry of Health. Women identified from the rosters were approached in their local health centers and invited to participate in the screening study. Women who were not present for their scheduled appointments were contacted and invited to return to the clinic during designated screening days, or were screened in their homes. Eligible participants were age 16 years or older, between 35 and 40 weeks of gestation based on self-reported date of last menstrual period or on last ultrasound reading (where available), resided in León, and planned to deliver in the public hospital in León. Enrolled women provided informed consent to provide health information and rectovaginal samples.

### 4.2. Data Collection and Handling, Transport, and Processing of Samples

During the health center visit, a trained study nurse or obstetrician-gynecologist administered a survey to enrolled women to collect a history of pregnancies and births, as well as risk factors for GBS colonization or infant GBS disease that were present in the current or a prior pregnancy. Infant GBS risk factors included prolonged rupture of membranes for >18 h; risk of preterm birth (defined as birth before 37 weeks gestation); risk of pregnancy loss (defined as vaginal bleeding before 20 weeks gestational age in the setting of a positive urine and/or blood pregnancy test with a closed cervical os, without passage of products of conception and without evidence of a fetal or embryonic demise); and fever ≥38 °C at any time during pregnancy. Urinary tract infection was considered a risk factor for maternal GBS colonization. The survey also collected gestational age at the time of screening based on ultrasound or the date of the last menstrual period when an ultrasound was not available. All data were self-reported by participants and later confirmed by a review of medical records. Some risk factors that might have been present at the time of delivery (i.e., PROM) were not captured during the screening visit. One-quarter of pregnant women had reached full term at the time of screening and could report complete information on earlier risk of preterm delivery.

**Figure 3:**
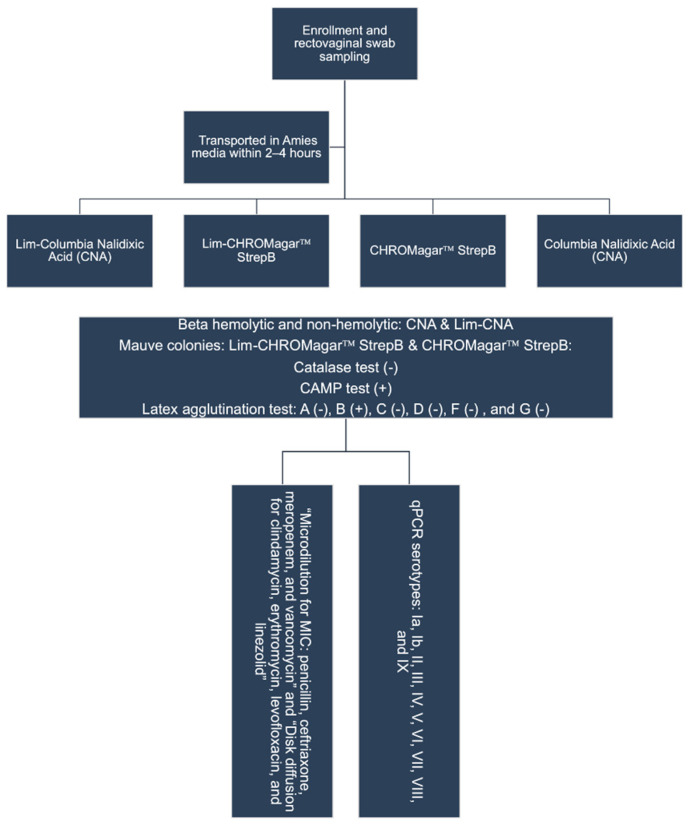
Study recruitment, sample collection, and laboratory analysis procedures.

Rectovaginal specimens were collected from pregnant women by a trained nurse or gynecologist, using one Remel BactiSwab Gel Collection and Transport Swab (Thermo Scientific™, Waltham, MA, USA) for each anatomical site. Swabs were placed in labeled Amies transport media with charcoal and transported to the laboratory of the Department of Microbiology at the Universidad Nacional Autónoma de Nicaragua, León within 4 hours of collection (Figure 3). 

### 4.3. Identification and Serotyping of Group B Streptococci

All specimens collected from pregnant women were analyzed following recommendations from the American College of Obstetricians and Gynecologists [2]. Rectovaginal swabs were cultured using: (1) direct plating onto CHROMagar™ StrepB; (2) direct plating onto Columbia Nalidixic Acid agar (CNA); and (3) incubation of samples in Lim broth followed by sub-culturing onto CHROMagar™ StrepB or CNA. After inoculation, CNA plates were incubated for 18–24 h at 37 °C in a CO_2_-rich atmosphere, while CHROMagar™ StrepB plates were incubated in aerobic conditions. If GBS was not detected, the CNA/CHROMagar™ StrepB plates were re-incubated and examined after 48 h to detect GBS. All colonies which were beta-hemolytic or non-hemolytic Gram-positive cocci, as well as catalase-negative cocci, were sub-cultured and isolated for confirmatory testing. An isolate which was CAMP-test positive was considered as suspected GBS, and later confirmed using a Strep B Grouping Latex Agglutination Kit (Thermo Scientific™) following the manufacturer’s recommendations, prior to qPCR serotype identification and antibiotic susceptibility testing. 

### 4.4. qPCR Serotyping

Primers and probes described by Breeding et al. to amplify regions of the polysaccharide capsular genes of *S. agalactiae* (Table 4) were used to further identify each of the GBS serotypes [32].

In brief, genomic DNA was extracted from overnight cultures of GBS ATCC reference strains (BAA-1138D-5™, BAA-1174™, BAA-2675™, BAA-2674™, BAA-2673™, BAA-611™, BAA-2671™, BAA-2670™, BAA-2669™, and BAA-2668™) and clinical isolates using the Qiagen DNeasy Blood and Tissue Kit according to the manufacturer’s instructions. qPCR reactions were performed in a final volume of 20 μL and consisted of 10 μL Bio-Rad iQ Multiplex Powermix (Bio-Rad Laboratories, Hercules, CA, USA), 7.0 μL sterile water, 0.5 μL forward primer (20 μM stock), 0.5 μL reverse primer (20 μM stock), 1.0 μL probe (20 μM stock), and 1 μL GBS DNA (25 ng/μL, unless otherwise indicated in the text). Triplicate reactions were performed on a LightCycler^®^ 96 Instrument (Roche Diagnostic, Mannheim, Germany). Reaction parameters were as follows: initial incubation at 50 °C for 3 min; initial denaturation at 95 °C for 10 min; 40 cycles of PCR at 95 °C for 15 s, and 60 °C for 1 min. Positive reactions were defined as a cycle threshold (CT) < 33 for 50 ng DNA template/reaction. Negative control reactions (no DNA template) were included with every run. Results were compared to latex agglutination. 

### 4.5. Antibiotic Susceptibility Testing

Procedures for disk diffusion testing for all clinical GBS isolates were performed in sextuplicate according to the methodologies described in the CLSI M100-ED31:2021 Performance Standards for Antimicrobial Susceptibility Testing, 31st Edition, for clindamycin, erythromycin, levofloxacin, and linezolid [33]. In addition, the minimum inhibitory concentrations (MICs) of penicillin G, ceftriaxone, meropenem, and vancomycin were determined by the microdilution method according to the CLSI’s recommendations, also in sextuplicate. Penicillin susceptibility was confirmed in duplicate in a random sample of 10 confirmed GBS isolates using e-test strips (Liofilchem^TM^ MTS^TM^) according to the manufacturer’s instructions. Additionally, qPCR was performed after the e-test to confirm the GBS serotype (Figure S2). *Streptococcus pneumoniae* ATCC 49619 was used as a control [33].

### 4.6. Statistical Analysis

We used descriptive statistics with chi-squared and Fisher’s exact tests to compare the clinical characteristics of women who were colonized with GBS to those who were not. We then used bivariate logistic regression models to estimate the odds ratios and 95% confidence intervals to describe the association between historical and current GBS risk factors. The distributions of GBS serotypes were described using proportions, as were the antimicrobial resistance or susceptibility of isolates to various antimicrobial agents. Correlations between penicillin MICs and resistance to other antimicrobial agents were estimated using linear regression with an R^2^ linear correlation coefficient. 

Statistical analyses were conducted using SAS v9.4 (SAS Institute, Cary, NC, USA) and GraphPad Prism 9.3.1 (GraphPad software, LLC) software. Antimicrobial susceptibility analysis was performed using WHONET software v. 21.13.26 (developed and supported by the WHO Collaborating Centre for Surveillance of Antimicrobial Resistance at the Brigham and Women’s Hospital in Boston, Massachusetts) [34].

## 5. Conclusions

In an urban area of Nicaragua, maternal GBS colonization reached 20%, which is concordant with global estimates. In addition, over 50% of GBS isolates showed resistance to first-line antimicrobial prophylaxis agents, and also high resistance to second-line agents. Nicaragua and surrounding countries with comparable GBS prevalence, serotype distribution, and resistance rates to commonly used IAP antibiotics would benefit from prophylactic maternal GBS immunization to deflect a large burden of infant GBS disease.

## Figures and Tables

**Table 1:** Characteristics of pregnant women recruited from public health centers and screened for rectovaginal GBS colonization in León, Nicaragua, 2019–2020 (N = 305).

Characteristics	Total (%) N = 305	GBS Colonization Detected		*p* ^a^
		Yes N = 63 (20.7%)	No N = 242 (79.3%)	
**Age**				0.036
Mean (±STD)	25.1 (± 6.14)	26.8 (± 6.69)	24.6 (± 5.91)	
<20 years	69 (22.6)	10 (15.9)	59 (24.4)	
20–24 years	83 (27.2)	15 (23.8)	68 (28.1)	
25–29 years	72 (23.6)	15 (23.8)	57 (23.6)	
30–34 years	56 (18.4)	12 (19.0)	44 (18.2)	
>35 years	25 (8.2)	11 (17.5)	14 (5.8)	
**Gestational age at screening**				
**By LMP ^b^** (N = 225)				
<37 weeks	168 (74.7)	37 (84.1)	131 (80.6)	0.47
37–41 weeks	57 (25.3)	10 (15.9)	47 (19.4)	
**By ultrasound ^b^** (N = 205)				
<37 weeks	176 (85.9)	41 (91.1)	135 (84.4)	0.34
37–41 weeks	29 (14.1)	4 (8.9)	25 (15.6)	
**Number of prior pregnancies**				0.12
0	117 (38.4)	22 (34.9)	95 (39.3)	
1	103 (33.8)	16 (25.4)	87 (36.0)	
2	55 (18.0)	18 (28.6)	37 (15.3)	
≥3	30 (9.8)	7 (11.1)	23 (9.5)	
**Number of prior births**				0.73
0	176 (57.7)	32 (50.8)	144 (59.5)	
1	82 (26.9)	21 (33.3)	61 (25.2)	
2–5	47 (15.5)	10 (15.9)	37 (15.3)	
**Number of prior pregnancy losses**				0.92
0	265 (86.9)	50 (79.4)	215 (88.8)	
≥1	40 (13.1)	13 (20.6)	27 (11.2)	
**Number of prior Cesarean deliveries**				0.11
0	246 (80.7)	50 (79.4)	196 (81.0)	
≥1	59 (19.3)	13 (10.6)	46 (19.0)	
**Pregnancy complications associated with maternal GBS colonization or infant GBS disease ^c^**				
**PROM > 18 h**	2 (0.7)	2 (3.2)	-	0.042
**Risk of preterm birth**	31 (10.2)	8 (12.7)	23 (9.5)	0.430
**Risk of pregnancy loss**	43 (14.1)	11 (17.5)	32 (13.2)	0.396
**Fever > 38 °C**	30 (9.8)	5 (7.9)	25 (10.3)	0.570
**Urinary tract infection**	85 (27.9)	15 (23.8)	70 (28.9)	0.410

Abbreviations; GBS = group B *Streptococci;* STD = standard deviation; LMP = last menstrual period; PROM = prolonged rupture of membranes. ^a^ Chi-squared test of proportions. ^b^ Fisher’s exact test used for cell sizes < 5. ^c^ PROM >18 h, risk of preterm birth, risk of pregnancy loss, and fever are associated with infant GBS disease; urinary tract infection is associated with maternal GBS colonization. Based on self-report and a confirmatory review of medical records.

**Table 2:** Association between pregnancy complications during a prior pregnancy and during the current pregnancy (n = 188).

Present in Current Pregnancy	Present in Prior Pregnancy n (%)		Odds Ratio (95% CI)	*p* ^a^
	Yes	No		
Prolonged rupture of membranes				
Yes (n = 1)	0 (0)	1 (100.0)	N/A	0.94
No (n = 187)	1 (0)	186 (100.0)		
Risk of pregnancy loss				
Yes (n = 35)	7 (20.0)	28 (80.0)	1.8 (0.7, 4.6)	0.24
No (n = 153)	19 (12.4)	134 (87.6)		
Risk of preterm birth				
Yes (n = 21)	4 (19.0)	17 (81.0)	3.6 (1.0–13.1)	0.03
No (n = 167)	10 (6.0)	157 (94.0)		
Fever > 38 °C				
Yes (n = 17)	1 (5.9)	16 (94.1)	1.5 (0.2–12.7)	0.72
No (n = 171)	7 (4.1)	164 (95.9)		
Urinary tract infection				
Yes (n = 49)	12 (24.5)	37 (75.5)	2.5 (1.1–5.7)	0.02
No (n = 139)	16 (11.5)	123 (88.5)		

Abbreviations: GBS = group B *Streptococci;* CI = confidence interval. ^a^ Based on self-report and a confirmatory review of medical records.

**Table 3:** Susceptibility and resistance of GBS isolates to eight antimicrobial agents (n = 85).

Antimicrobial	MIC_50_	MIC_90_	MIC Range	Resistant ^c^ n (%)	Intermediate ^d^ n (%)	Susceptible ^e^ n (%)
Clindamycin ^a^	N/A	N/A	N/A	27 (31.7)	6 (7.1)	52 (61.2)
Erythromycin ^a^	N/A	N/A	N/A	32 (37.6)	19 (22.4)	34 (40.0)
Penicillin ^b^	0.25	2	0.032–4.00	45 (52.9)	0	40 (47.1)
Ceftriaxone ^b^	0.064	2	0.032–2.00	21 (24.7)	0	64 (75.3)
Meropenem ^b^	0.064	2	0.064–2.00	18 (21.2)	0	67 (78.8)
Levofloxacin ^a^	N/A	N/A	N/A	0	1 (1.2)	84 (98.8)
Linezolid ^a^	N/A	N/A	N/A	1 (1.2)	0	84 (98.8)
Vancomycin ^b^	0.5	1	0.25–2.00	8 (9.4)	0	77 (90.6)

^a^ Testing was performed by disk diffusion. ^b^ Testing was performed by microdilution. ^c^ Resistant, ^d^ Intermediate, and ^e^ Susceptible as defined in CLSI M100-ED31:2021 Performance Standards for Antimicrobial Susceptibility Testing, 31st Edition. N/A: Not applicable.

**Table 4:** Target gene sequences, primers, and probes for GBS serotyping by real-time polymerase chain reaction ^a^.

Serotype	Sequence (5′–3′)	Gene Target	Size (bp)
**Ia-F**	GTTTAAAAATCCTGATTTTGATAGAATTTTAGCAGCTTTTAAC	*cpsH*	207
**Ia-R**	CTGATATTTTGAATATTATTATGCAAACAATAATAATATGTTCCCCCTA		
**Ia-P**	6-FAM-TCGTTGATT/ZEN/ATCGGTATAGTATCATTG GCT-IAbFQ		
**Ib-F**	GTATTAAATTCGTTATTTAGAAGTCCAGAATTTCATAGAGTCATTGC	*cpsH*	195
**Ib-R**	GGCATAATAATATAGAAATCCTAAACAAGACAAAATAATTGCATTAAAC		
**Ib-P**	6-FAM-TGC ATT CAA/ZEN/TTCACTGGCAGTAGGG- IAbFQ		
**II-F**	CACATATATATTAAAGTTCACCCTAGAGATAACATTGACTACTCTAATC	*cpsK*	151
**II-R**	CTAATGCCGTGGAAAAATATGTAATCCCAACATCAAATT		
**II-P**	6-FAM-AATGCAACA/ZEN/GTAATACAAAGGAACATC CCT- IAbFQ		
**III-F**	GGAATTGTTCTTTATTTTTCTGCCT	*cpsI*	170
**III-R**	ACTATACCAAAAGTTGAGAATAATAATACAATACTCCAATGA		
**III-P**	6-FAM-ATGTTACAC/ZEN/GCTCTTTGAGGAAATAGATCC- IAbFQ		
**IV-F**	GAAGAAAATATATATTTGCCATACAGTATATCATCTCCTTATTACAATTATCA	*cpsK*	159
**IV-R**	CATAGAATACCTTCTTTATTGGTACGTTTACATAAATCATCAATATTAAC		
**IV-P**	6-FAM-AGGGAACAG/ZEN/AGGAGATCAATAATTATATTGGC- IAbFQ		
**V-F**	CAAAATTCAATGAGAGAATGTTGTATTTTTTTGAGGCAATTC	*cpsO*	153
**V-R**	CAATCATCTTCCCACATATATCTATTCCACCAAATACTTC		
**V-P**	6-FAM-ATTTTCCAC/ZEN/ATAATACATCTTTAATCTCTGCTG T- IAbFQ		
**VI-F**	GACAGTCTATTACGAAAGTATAAGAGCGATT	*cpsH*	219
**VI-R**	AGCTTGTAGATTATCCTGTTTTGTTTGATAGCTTCTCTATATAG		
**VI-P**	6-FAM-CCCTCCAGT/ZEN/GTGGGAATATTTTTAGGTTCAC- IAbFQ		
**VII-F**	GAGGGCTTACCTCACGACAGGAGAAGTAAAAAATATAAAG	*cpsK*	160
**VII-R**	GCTGCGTTAATAACAATACTGACTTTGGAGC		
**VII-P**	6-FAM-AGTCTTACC/ZEN/CAAGAACAAAAGTCTCTGATT- IAbFQ		
**VIII-F**	GACTAATGGTTAAGTATGCTAACTTGCTAATTTGTGATAGTAA	*cpsR*	152
**VIII-R**	CTTGTCCTTAAAATTGTGTTTTGACTTTGTCAGATCAGTC		
**VIII-P**	6-FAM-ATGCTCCTA/ZEN/AAACAACCTACATCGCCTATG- IAbFQ		
**IX-F**	CATTGAGCAAAGAGAAAACAGTATATGTCAAAGGGC	*cpsO*	128
**IX-R**	ATGTTCAAGGATAAAATCTCTATTATGTTGCATTGCTTCA		
**IX-P**	6-FAM-AGTACTACC/ZEN/AGACAGTCATACAAAGAGAAT- IAbFQ		

Sequences are presented in the 5′ to 3′ direction with probe modifications as indicated (6-carboxyfluorescein [6-FAM] fluorescent probe, internal ZENTM quenchers, and Iowa Black^®^ FQ [IAbFQ]). ^a^ [32].

## Data Availability

Data are available from the investigators on request and on approval of a data use agreement.

## Supplementary Materials

The following supporting information can be downloaded at: https://www.mdpi.com/article/10.3390/pathogens11040415/s1. Figure S1: Group B *Streptococci* recovery by culture method (n = 91); Figure S2: Comparison of penicillin minimum inhibitory concentration (MIC) values determined by microdilution and e-test strips on 10 randomly selected Group B Streptococcus (GBS) isolates. Shown here is an example of an e-test result from a plated GBS isolate.
