# Supplementary material for: Rectovaginal Colonization with Serotypes of Group B Streptococci with Reduced Penicillin Susceptibility among Pregnant Women in León, Nicaragua
Source: Pathogens. 2022 Mar 29;11(4):415. doi: 10.3390/pathogens11040415 (PMC9029029; doi:10.3390/pathogens11040415)
Supplement: Supplementary file 1 [file pathogens-11-00415-s001.zip › pathogens-1639310-supplementary.pdf]

Figure S1. Group B *Streptococcus* recovery by culture method (n=91)

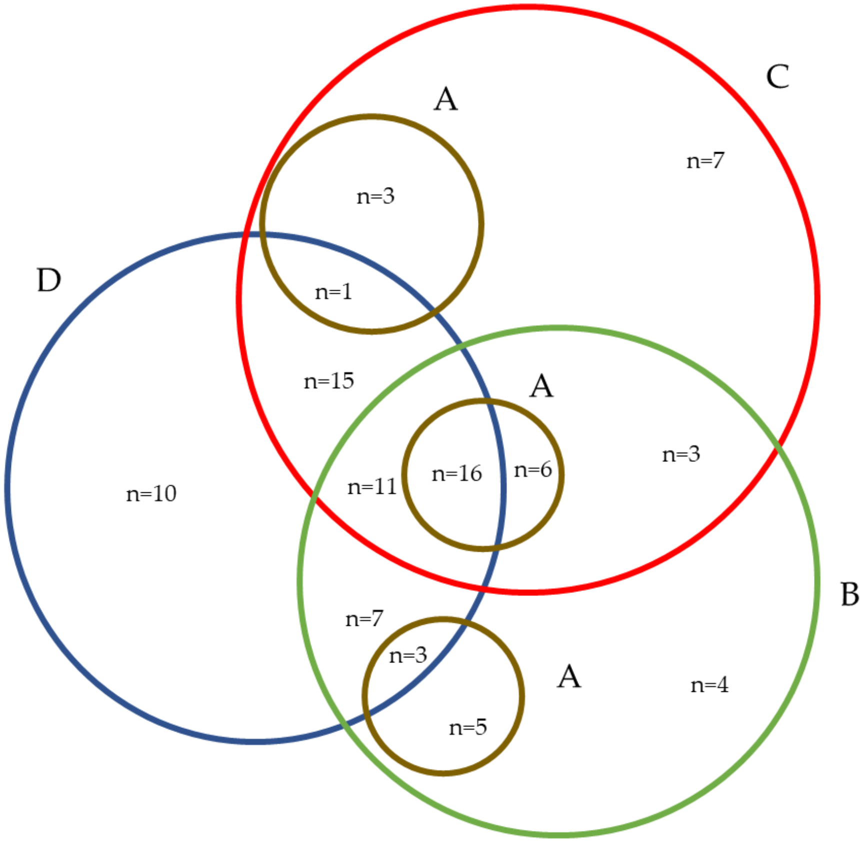

Culture method	# samples with GBS recovery
Lim+CHROMagar, Lim+CNA, CHROMagar, CNA	16
Lim+CHROMagar, CHROMagar	15
Lim+CHROMagar, Lim+CNA, CHROMagar	11
Lim+CHROMagar	10
Lim+CHROMagar, Lim+CNA	7
CHROMagar	7
Lim+CNA, CHROMagar, CNA	6
Lim+CNA, CNA	5
Lim+CNA	4
Lim+CHROMagar , Lim+CNA, CNA	3
Lim+CNA, CHROMagar	3
CHROMagar, CNA	3
Lim+CHROMagar, CHROMagar, CNA	1

A=Columbia Nalidixic Acid (CNA; N=34)
B=LIM+CNA (N=55)
C=CHROMagar StrepB (N=62)
D=LIM+CHROMagar StrepB(N=63)
